# Supplementary material for: Aorto-Iliac Artery Calcification and Graft Outcomes in Kidney Transplant Recipients
Source: J Clin Med. 2021 Jan 17;10(2):325. doi: 10.3390/jcm10020325 (PMC7829792; doi:10.3390/jcm10020325)
Supplement: Supplementary file 1 [file jcm-10-00325-s001.pdf]

Journal of
Clinical Medicine

Aorto-Iliac Artery Calcification and Graft Outcomes in Kidney Transplant Recipients

Supplementary Material

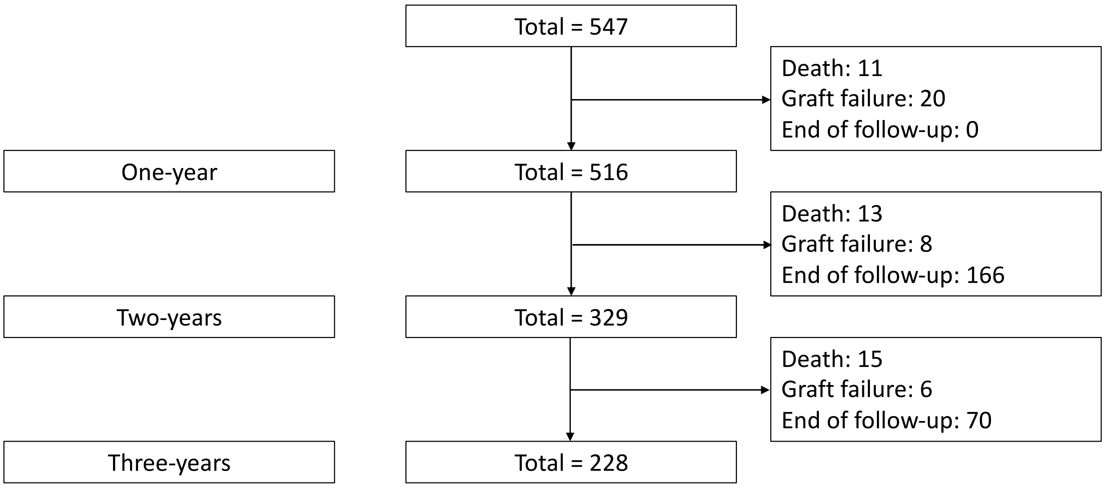

Figure S1. Flowchart for death, graft failure and end of follow-up in the first three years after kidney transplantation.

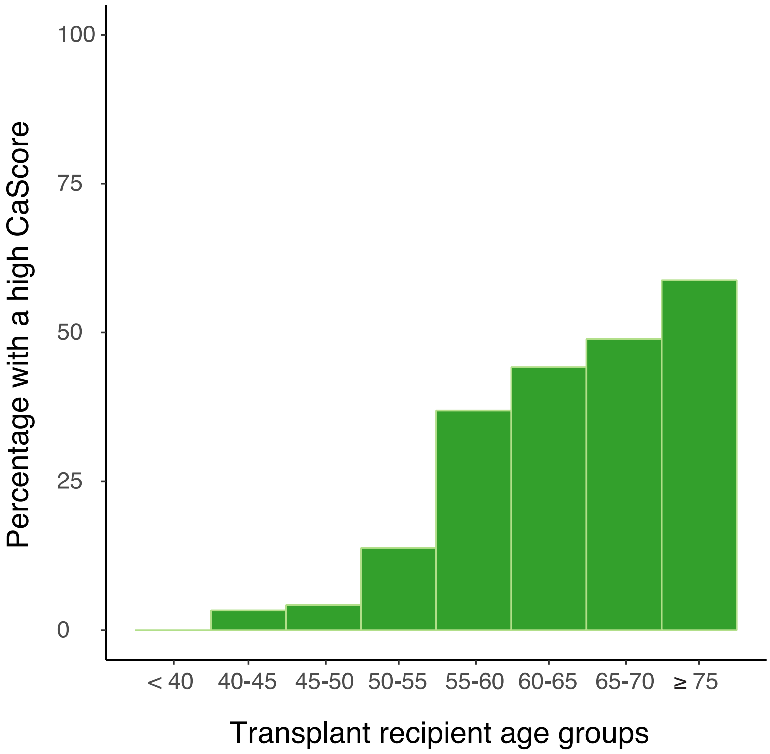

Figure S2. Distribution of patients with a high CaScore for eight age groups.

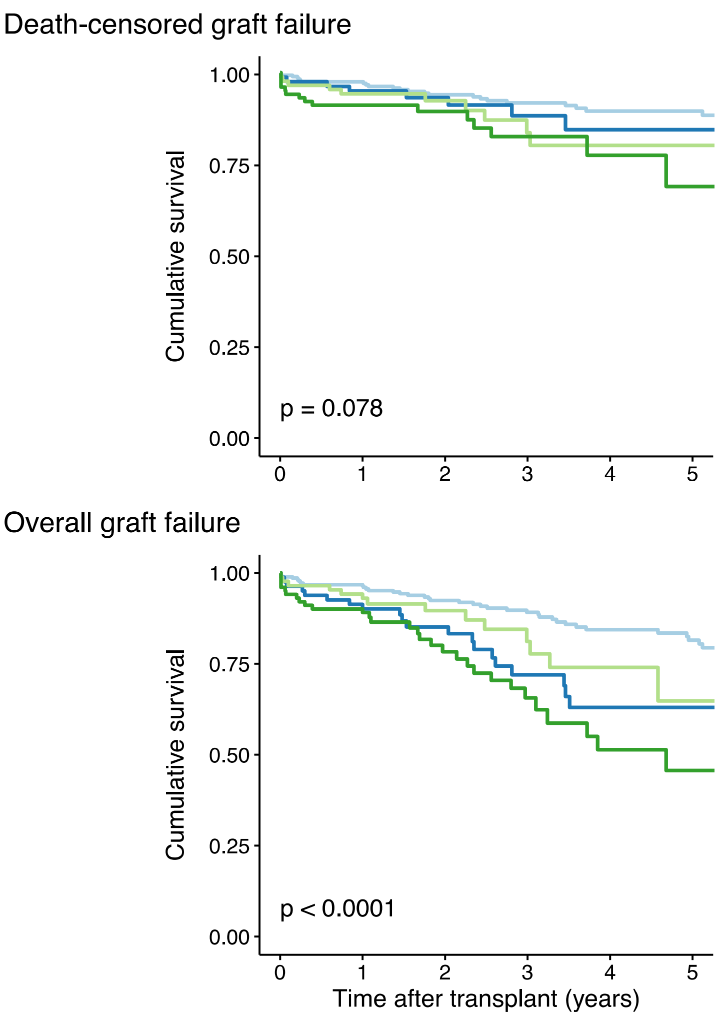

No. at risk

< 65 – low CaScore	279	270	193	150	104	82
< 65 – high CaScore	81	74	48	30	19	13
≥ 65 – low CaScore	86	81	43	24	13	7
≥ 65 – high CaScore	101	91	45	24	12	7

Figure S3. Kaplan-Meier survival curve for death-censored graft failure and overall graft failure free-survival for the low and high aorto-iliac CaScore group, stratified by transplant recipient age (< 65 or ≥ 65 years-of-age), including a life table applicable for both graphs. Number at risk (No. at risk) provided for all four groups.

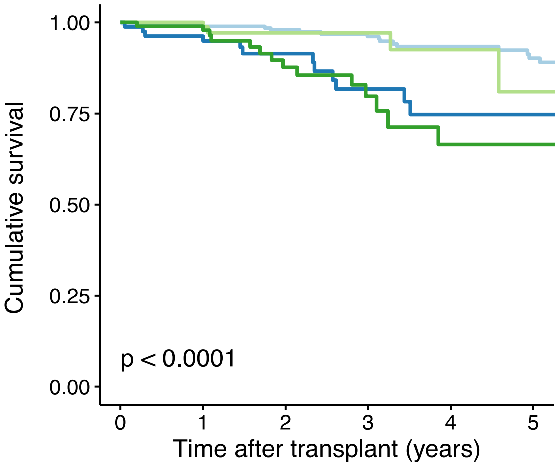

No. at risk

< 65 – low CaScore	279	270	193	150	104	82
< 65 – high CaScore	81	74	48	30	19	13
≥ 65 – low CaScore	86	81	43	24	13	7
≥ 65 – high CaScore	101	91	45	24	12	7

Figure S4. Kaplan-Meier survival curve for death with a functioning graft free-survival for the low and high aorto-iliac CaScore group stratified by transplant recipient age (< 65 or ≥ 65 years-of-age). Number at risk (No. at risk) provided for all four groups.

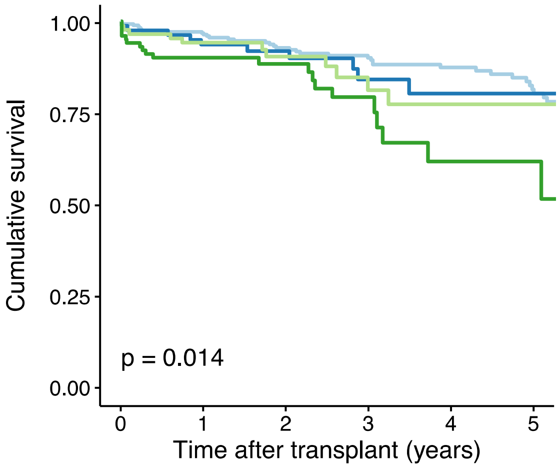

No. at risk

< 65 – low CaScore	279	267	190	148	103	76
< 65 – high CaScore	81	72	48	29	19	13
≥ 65 – low CaScore	86	81	42	23	13	7
≥ 65 – high CaScore	101	89	45	23	10	6

Figure 5. Kaplan-Meier survival curve for graft function decline free-survival for the low and high aorto-iliac CaScore group stratified by transplant recipient age (< 65 or ≥ 65 years-of-age). Number at risk (No. at risk) provided for all four groups.

Table S1. Characteristics stratified by donor status.

Variables	Living-donation (n = 301)	Deceased-donation (n = 246)	p-value
**Patient**			
Male gender, *n (%)* [a]	187 (62.1)	149 (39.4)	0.776 [b]
Age, *years* [c]	58 [51, 65]	64 [51, 70]	**<0.001** [d]
Diabetes mellitus, *n (%)* [a]	68 (22.6)	105 (42.7)	**<0.001** [b]
Body Mass Index, *kg/m*^{2} [b]	26.7 (4.7)	27.0 (4.7)	0.457 [c]
Smoker, *n (%)* [a]			0.343 [b]
Non	79 (26.2)	75 (30.5)	
Former	191 (63.5)	141 (57.3)	
Current	31 (10.3)	30 (12.2)	
Hypercholesterolemia, *n (%)* [a]	86 (28.6)	67 (27.2)	0.802 [b]
Total cholesterol, *mmol/L*	4.6 (1.2)	4.8 (1.4)	0.240 [c]
Systolic blood pressure, *mmHg*	145 (20.)	145 (25)	0.850 [c]
Use of antihypertensive medication, *n (%)*	233 (77.4)	206 (83.7)	0.081 [b]
Dialysis vintage, *months* [c]	0 [0, 14]	25 [13, 43]	**<0.001** [d]
Previous transplants, *n (%)* [a]			0.426 [b]
Non	284 (94.4)	230 (93.5)	
One	13 (4.3)	9 (3.7)	
Two	4 (1.3)	7 (2.8)	
Aorto-iliac CaScore, *HU*	2163 [209, 6331]	4037 [505, 8616]	**0.006** [d]
**Transplantation**			
Male gender donor, *n (%)*	146 (48.5)	148 (60.2)	**0.008** [b]
Donor age, *years*	54 (12)	54 (16)	0.571 [c]
No. of HLA-mismatches, *n*	3.6 (1.5)	3.3 (1.5)	**0.013** [c]
Warm ischemia time, *minutes*	40 (21)	43 (49)	0.348 [c]
Cold ischemia time, *minutes*	169 (64)	756 (248)	**<0.001** [c]
**Follow-up** [e]			
eGFR at six-months [a]	51 (17)	48 (20)	**0.037** [c]
eGFR at one-year [a]	54 (18)	47 (23)	**<0.001** [c]
Haemoglobin at one-year, *mmol/L*	8.2 (1.1)	8.0 (1.0)	0.076 [c]
Calcium at one-year, *mmol/L*	2.44 (0.13)	2.43 (0.15)	0.458 [c]
Phosphate at one year, *mmol/L*	0.93 (0.21)	0.93 (0.21)	0.652 [c]
Albumin at one-year, *g/L*	44 (3)	43 (3)	**<0.001** [c]
Glucose at one-year, *mmol/L*	5.7 [5.1, 6.9]	6.1 [5.2, 8.3]	**0.004** [d]
PTH at one-year, *pmol/L*	9 [7, 14]	12 [9, 17]	**<0.001** [d]
Protein excretion at one-year, *g/24h*	0.2 [0.1, 0.2]	0.2 [0.1, 0.3]	**0.045** [d]
Cytomegalovirus infection, *n (%)*	7 (2.3)	22 (8.9)	**<0.001** [b]

HU = Hounsfield units; HLA = human leukocyte antigen; eGFR = estimated-Glomerular Filtration Rate; PTH = parathyroid hormone; [a] eGFR – CKD-EPI (Chronic Kidney Disease Epidemiology Collaboration) (mL/min per 1.73 m2); [b] p-value by chi-square test; [c] p-value by student-t test; [d] p-value by Mann-Whitney U test; [e] data available for 462 patients

Table S2. Multivariable adjusted associations of the CaScore with one-year eGFR after multiple imputation by chained equations.

	Standard β	95% CI	p-value
**One-year eGFR**			
Univariate	-3.3	-5.1 to -1.5	**<0.0001**
Model 1	-3.0	-4.8 to -1.2	**<0.0001**
Model 2	-3.1	-4.9 to -1.3	**<0.0001**
Model 3	-1.5	-3.6 to 0.6	0.160
Model 4	-0.7	-2.7 to 1.2	0.271

Linear regression analysis after multiple imputation by chained equations (MICE). Data are presented as hazard ratio and 95% confidence interval (CI) for the continuous CaScore (natural log transformed). Model 1: adjusted for transplant centre and time between computed tomography and transplantation; model 2: adjusted for model 1 plus donor gender, donor type (living donation, DCD or DBD), cold ischemia time, no. of HLA mismatches, recipient gender, diabetes mellitus, smoking, dialysis vintage, no. of previous transplantations, statin use, laboratory results at one year after transplantation (serum phosphate, serum calcium, serum glucose, serum haemoglobin, serum PTH, proteinuria), and episode of acute rejection; model 3: adjusted for model 2 plus recipient age; model 4: adjusted for model 3 plus donor age

Table 3. Multivariable adjusted associations of the CaScore with one-year eGFR.

	**Living-donation**			**Deceased-donation**		
	Standard β	95% CI	p-value	Standard β	95% CI	p-value
**One-year eGFR**						
Univariate	-1.9	-4.1 to 0.2	0.079	-4.6	-7.5 to -1.6	**0.002**
Model 1	-2.3	-4.5 to -0.1	**0.041**	-4.3	-7.3 to -1.3	**0.005**
Model 2	-4.0	-6.1 to -1.9	**<0.001**	-4.6	-7.7 to –1.5	**0.004**
Model 3	-2.1	-4.5 to 0.2	0.070	-3.7	-7.2 to -0.1	**0.049**
Model 4	-1.6	-3.9 to 0.6	0.149	-1.9	-5.3 to 1.6	0.287

Linear regression analysis, data available for 462 patients in total. Data are presented as hazard ratio and 95% confidence interval (CI) for the continuous CaScore (natural log transformed). Model 1: adjusted for transplant centre and time between computed tomography and transplantation; model 2: adjusted for model 1 plus donor gender, cold ischemia time, no. of HLA mismatches, recipient gender, diabetes mellitus, smoking, dialysis vintage, no. of previous transplantations, statin use, laboratory results at one year after transplantation (serum phosphate, serum calcium, serum glucose, serum haemoglobin, serum PTH, proteinuria), and episode of acute rejection; model 3: adjusted for model 2 plus recipient age; model 4: adjusted for model 3 plus donor age

Table S4. Multivariable adjusted associations of the CaScore with (death-censored) graft failure, death with a functioning graft and graft function decline.

	**Living-donation** (n = 301)				**Deceased-donation** (n = 246)			
	Low CaScore	High CaScore			Low CaScore	High CaScore		
	Hazard ratio	Hazard ratio	95% CI	p-value	Hazard ratio	Hazard ratio	95% CI	p-value
**Death censored graft failure**								
Univariate	1.0 (Ref)	0.8	0.3 to 2.3	0.639	1.0 (Ref)	1.7	0.9 to 3.2	0.088
Model 1	1.0 (Ref)	0.9	0.3 to 2.8	0.894	1.0 (Ref)	1.7	0.9 to 3.1	0.096
Model 2	1.0 (Ref)	0.9	0.3 to 3.2	0.982	1.0 (Ref)	1.7	0.9 to 3.3	0.100
Model 3	1.0 (Ref)	0.8	0.2 to 2.7	0.740	1.0 (Ref)	1.3	0.6 to 2.9	0.442
Model 4	1.0 (Ref)	0.8	0.2 to 2.7	0.730	1.0 (Ref)	1.3	0.6 to 2.7	0.458
**Overall graft failure**								
Univariate	1.0 (Ref)	2.0	1.1 to 3.6	**0.020**	1.0 (Ref)	2.6	1.6 to 4.2	**<0.001**
Model 1	1.0 (Ref)	2.2	1.2 to 4.0	**0.011**	1.0 (Ref)	2.6	1.6 to 4.2	**<0.001**
Model 2	1.0 (Ref)	2.2	1.3 to 4.7	**0.007**	1.0 (Ref)	2.6	1.6 to 4.3	**<0.001**
Model 3	1.0 (Ref)	2.0	1.0 to 3.9	**0.047**	1.0 (Ref)	1.8	1.0 to 3.2	**0.047**
Model 4	1.0 (Ref)	2.0	1.1 to 4.2	**0.036**	1.0 (Ref)	1.8	1.0 to 3.2	**0.048**
**Death with a functioning graft**								
Univariate	1.0 (Ref)	3.2	1.5 to 6.5	**0.002**	1.0 (Ref)	4.9	2.2 to 11.0	**<0.001**
Model 1	1.0 (Ref)	3.1	1.5 to 6.6	**0.003**	1.0 (Ref)	4.8	2.1 to 10.9	**<0.001**
Model 2	1.0 (Ref)	3.7	1.7 to 8.6	**0.002**	1.0 (Ref)	5.0	2.1 to 12.0	**<0.001**
Model 3	1.0 (Ref)	2.9	1.2 to 7.0	**0.017**	1.0 (Ref)	4.9	1.9 to 12.3	**<0.001**
Model 4	1.0 (Ref)	3.1	1.2 to 7.6	**0.015**	1.0 (Ref)	2.8	1.0 to 7.8	**0.042**
**Graft function decline**								
Univariate	1.0 (Ref)	0.9	0.4 to 2.1	0.825	1.0 (Ref)	2.0	1.1 to 3.5	**0.021**
Model 1	1.0 (Ref)	1.0	0.4 to 2.5	0.919	1.0 (Ref)	2.0	1.1 to 3.5	**0.022**
Model 2	1.0 (Ref)	1.0	0.4 to 2.7	0.882	1.0 (Ref)	2.1	1.1 to 3.9	**0.019**
Model 3	1.0 (Ref)	0.8	0.3 to 2.2	0.792	1.0 (Ref)	1.6	0.8 to 3.1	0.192
Model 4	1.0 (Ref)	0.8	0.4 to 2.4	0.877	1.0 (Ref)	1.6	0.9 to 3.5	0.132

Cox proportional hazards regression analysis. Data are presented as hazard ratio and 95% confidence interval (CI) for the two aorto-iliac CaScore groups (low and high). Model 1: adjusted for transplant centre and time between computed tomography and transplantation; model 2: adjusted for model 1 plus donor gender, cold ischemia time, no. of HLA mismatches, recipient gender, diabetes mellitus, dialysis vintage, and no. of previous transplantations, statin use; model 3: adjusted for model 2 plus recipient age; model 4: adjusted for model 3 plus donor age.
